# Impact of COVID-19 on Rocky Vista University medical students’ mental health: A cross-sectional survey

**DOI:** 10.3389/fpsyg.2023.1076841

**Published:** 2023-02-06

**Authors:** Dean C. Paz, Manav Singh Bains, Morgan L. Zueger, Varasiddimounish R. Bandi, Victor Y. Kuo, Mark Payton, Rebecca Jean Ryznar

**Affiliations:** Rocky Vista University, Parker, CO, United States

**Keywords:** medical students, COVID-19, Coronavirus, anxiety, depression, mental health, mood, anxiety disorder

## Abstract

**Background:**

The COVID-19 pandemic brought immense changes to medical school curriculums world-wide, such as the widespread adoption of virtual learning. We sought to better understand the impact on medical students’ mental health at Rocky Vista University College of Osteopathic Medicine, Parker, CO, United States. This study assessed the impact the pandemic had on anxiety and depression levels of medical students. It also assessed the impact of several domains on student mental health during the pandemic and how various sub-groups within the studied population were affected.

**Methods:**

A cross-sectional survey was sent to students through an online anonymous google survey in May to June 2021, centered around the 7-item questionnaire used to screen for Generalized Anxiety Disorder GAD-7, 9-item questionnaire used to screen for depression PHQ-9, and self-designed questions to assess the personal impact of the pandemic. Data obtained were screened for error and analyzed with significance value of *p* < 0.05.

**Results:**

A total of 152 responses were received (25.5% response rate). Of these, 64.1% identified as female, 75.8% were white, 50.3% were between ages 21–25, and 77.8% were first year medical students. During the pandemic, 79.6% of respondents felt more anxious and 65.1% felt more depressed. 67.8% of students reported feeling social isolation amidst the pandemic. Students living with friends were more likely to see a therapist for depression during the pandemic (*p* = 0.0169) and prescribed an antidepressant (*p* = 0.0394). Females and students in relationships were more likely to score higher on GAD-7 (*p* = 0.0194) and (*p* = 0.0244), respectively.

**Conclusion:**

This study investigated the effect of the pandemic on medical students’ mental health and the need to address this issue. Results suggest that the pandemic had a negative impact on medical student’s mental health and that anxiety and depression levels worsened for pre-clinical medical students at Rocky Vista University. As such, it is imperative to incorporate additional resources to protect the well-being of medical students as they progress through their medical careers.

## Introduction

The first human cases of COVID-19 (SARS-CoV-2) were reported in December 2019 by officials of Wuhan City, China (World Health Organization, 2020). On 11 March 2020, the WHO declared the novel coronavirus outbreak a global pandemic and recommendations of social distancing, quarantining and isolation were emphasized and enforced (Cucinotta and Vanelli, 2020; Sohrabi et al., 2020). Educational institutions responded with necessary adaptations, including the radical transformation from in-person learning to virtual curriculums by preclinical medical schools (O’Byrne et al., 2020; Woolliscroft, 2020; Kaul et al., 2021). The longevity of such isolating measures during the pandemic have placed a high burden on the mental health of many populations (Department of Microbiology, Quaid-i-Azam University, Islamabad, Pakistan, Anjum S, Ullah R, Department of Microbiology, Sarhad University of Science and Information Technology, Peshawar, Pakistan, Suleman Rana M, Department of Virology, National Institute of Health, Islamabad, Pakistan, 2020; Hossain et al., 2020). Such isolation from the restriction, has been shown to affect academic performance in university students, along with negative exacerbation of their mental health symptoms (Giusti et al., 2021). Many aspects that play a role in the quality of life for students have been impacted by the pandemic and these have been shown to influence the mental health of university students and medical students as well (Giusti et al., 2020; Carletto et al., 2022). In particular, medical students face a higher level of demand and stress than most at baseline, as medical education is considered to be among the most academically challenging and emotionally demanding of higher education programs (Rotenstein et al., 2016). This has been implicated in having a negative effect on medical student mental health and may exacerbate the increasing prevalence of physician burnout and growing shortage of physicians in the United States (Mosley et al., 1994; Dyrbye et al., 2006; Tian-Ci Quek et al., 2019). We found few studies in assessing United States medical student mental health, though there were numerous articles recognizing the need for improvement (Slavin et al., 2014; Harries et al., 2021). Negative mental health symptoms, which have been found to be exacerbated by the COVID-19 pandemic, have been shown to reduce cognitive empathy levels in medical students (Peifer and Taasoobshirazi, 2022). Being able to express cognitive empathy with patients and relatives is important as it allows for the ability to deliver proper care and has been shown to result in better health outcomes (Giusti et al., 2021).

The research analyzing baseline mental health data, prior to the pandemic, for the medical student population living in the United States, is seriously lacking. To find recent baseline anxiety prevalence data using the GAD-7, we expanded our search outside of the United States to include the global medical student population. A meta-analysis was conducted in 2019, prior to the pandemic, revealing a global anxiety prevalence of 33.8% (Tian-Ci Quek et al., 2019). Baseline depression prevalence data using the PHQ-9 was more accessible but potentially outdated; a study conducted in Michigan will be used to compare pre- and post-pandemic rates. Their analysis of medical student depression in 2010 revealed a prevalence of moderate to severe depression of 14.3% (Nkire et al., 2021). As we did not have this data for our specific population from before the pandemic, we will be using such previous data for baseline reference while conducting our study to analyze the changes in mental health.

We have found substantially more research done on international medical student populations, especially projects that were completed during the pandemic. In general, these studies found that pandemic control measures detrimentally impacted medical student mental health (Aebischer et al., 2020; Loda et al., 2020; Saddik et al., 2020; Xiao et al., 2020; Xie et al., 2020; Essangri et al., 2021; Halperin et al., 2021; Nishimura et al., 2021). However, such studies are lacking in the United States. Given this dearth of mental health research on United States medical student populations and the potential magnifying impact of COVID-19 changes, we felt it was paramount to assess the mental health of preclinical medical students (1st and 2nd years of medical school) during the pandemic.

Our study, conducted a year after COVID-19 lockdown measures were first implemented, reports on the anxiety and depression levels of preclinical medical students at Rocky Vista University. In addition, we explored specific characteristics that may have contributed to such levels. We believe that the COVID-19 pandemic has had a negative effect on the mental health of pre-clinical medical students at Rocky Vista University College of Osteopathic Medicine in Parker, CO, United States (RVU-COM) and this has led to a worsening in anxiety and depression levels. Our study is also seeking to explore the role of various demographic factors in potentially impacting the mental health of medical students, in relation to the COVID-19 pandemic. Along with this, we sought to assess other subjective factors to get a better understanding on how the thought process of medical students is being affected. These are factors such as utilization of mental healthcare, changes in wellness behaviors, perceived effect of the pandemic on medical education, willingness of the medical student to clinically assist in the pandemic and their perception on the personal risk of infection. These factors will provide us with a better understanding of how the pandemic has affected the medical students and how these factors could be influencing their mental health. These are important factors to assess, as medical students are the future care providers and mental health has been shown to affect care if not properly addressed (Søvold et al., 2021). We evaluated various sub-groups within our study population, such as gender, class year, age range, relationship status, living situation and campus. We felt that these subgroups had a significant impact on student lives, namely living situation, marital status, age range, and class year. Through our study, we were striving to assess any mental health disparities that might exist within these sub-groups.

## Methods

We conducted a cross-sectional survey about a year after the initial lockdowns around the United States, from May to June 2021. Surveys were sent over social media channels, including GroupMe and Microsoft Teams, to OMS-I and OMS-II, first and second year students, respectively, on both Rocky Vista University College of Osteopathic Medicine (RVU) campuses located in Parker, Colorado and Ivins, Utah. All OMS-I and OMS-II students enrolled at RVU were eligible. Reminders to complete the survey were sent halfway into and at the end of the survey period *via* the above general social media channels without the use of personal information. Traditional undergraduate medical education in the United States consists of 2 years of pre-clinical classroom-based curriculums followed by 2 years of clinical training in hospital and outpatient settings. Prior to the pandemic, RVU followed this model with primarily in-person classes and skills labs. RVU switched to a completely virtual curriculum at the beginning of the pandemic for the remainder of the 2020 spring semester beginning in late March and lasting for the remainder of that semester, finishing in June 2021. The 2020–2021 academic year curriculum consisted of a hybrid of primarily online classes, with weekly in-person skills labs. The majority of OMS-II students who participated in the survey began their medical education in July 2019 with planned graduation in 2023. The majority of OMS-I students who participated in the survey began in July 2020 with planned graduation in 2024. OMS-III and OMS-IV students were excluded to assess the impact of hybrid educational curricula on mental health. Only medical students who were currently enrolled at the time of survey distribution were included. This study was approved by the RVU Institutional Review Board, IRB #2021–0001.

We developed a survey instrument centered around GAD-7, a validated 7-item questionnaire used to screen for Generalized Anxiety Disorder, and PHQ-9, a validated 9-item questionnaire used to screen for depression, as described by DSM-IV (Kroenke et al., 2001; Spitzer et al., 2006). The survey instrument also included self-designed questions intended to assess the impact of the several domains on student mental health during the pandemic, including: student demographics, perceived effect on medical education, willingness to clinically assist with the pandemic, perceptions of personal risk of infection, changes in wellness behaviors, and usage of mental healthcare. After creation, all authors participated in a multi-stage review process to optimize the content, clarity, intent, and validity of questions included in the survey to minimize self-reporting bias. The final instrument consisted of 82 questions. Questions included were primarily administered on a 5-point Likert scale (1 = strongly disagree to 5 = strongly agree), with a minority of yes/no, multiple choice, and free response questions. The full survey can be found in Supplementary material (Survey Instrument). Surveys were distributed over the course of 1 month (18 May 2021 to 04 June 2021) by social media channels and email, with periodic follow-up messages to increase response rate. Surveys were conducted anonymously.

Survey data was managed using Google Forms. All surveys were assessed for validity and completeness. All surveys were greater than 90% completed and therefore included. For data analysis, SAS (Version 9.4, SAS Institute, Cary, NC) was used. Participant characteristics and response results for key questions were summarized as raw counts and frequency percents. Responses to Likert scale questions were reported as combined positive responses (agree or strongly agree) and negative responses (disagree or strongly disagree) and breakdowns for each response. PHQ-9 and GAD-7 results were reported as raw scores and frequency percents. To assess associations of GAD-7 and PHQ-9 scores and key questions by subgroup, we used correlation analyses, ANOVA, and t-tests with a *p-*value of <0.05 to show significance. Initial analyses in Tables 1, 2 were conducted using ANOVA tests unless otherwise noted. In Table 2, Likert groupings were paired according to the following: groups 1 and 2, and groups 3–5. This was done in order to simply and increase the strength of the data. *Post-hoc* ANOVA analyses for tests with significant Chi-square values were performed using Benjamini–Hochberg procedures.

**Table 1:** Association of demographics with GAD-7 and PHQ-9 scores.

Association of anxiety and depression scores and demographic factors				
	Mean GAD-7 score	*p*-value	Mean PHQ-9 score	*p*-value
Gender		0.0194		0.8499
Female	9.03		7.57	
Male	6.92		7.3962	
Class year		0.3868		0.0142
OMS I	8.05		6.83	
OMS II	8.94		9.5	
Age (correlation analysis)	*r* = −0.0173	0.8335	*r* = 0.09	0.25
Relationship status		0.0244		0.9901
Single	7.48		7.34	
In a relationship	9.5		7.48	
Married/engaged	6.81		7.48	
Living situation		0.3157		0.4911
With friends	9.2		8.53	
With FAMILY	8.89		6.94	
Alone	7.28		6.9	
Significant other	8.08		7.2	
Significant other w/children	4.75		4.5	
Campus		0.8205		0.0648
CO	8.18		6.81	
SU	8.39		8.77	
Ethnicity		0.3487		0.5441
Asian	7.18		7	
Black	3.5		2.5	
Latino	6.43		5.43	
Mixed	7		5.67	
Native	14		12	
White	8.72		7.83	

**Table 2:** Association of anxiety and depression questions with demographics.

Likert questions association with demographics		
	Chi-Square coefficient	*p*-value
**I have felt more anxious during the COVID-19 pandemic**		
Gender	4.5324	0.0333*
Class year	2.5484	0.636
Age (ANOVA)	−1.1	0.28
Relationship status	0.1779	0.9149
Living situation	0.8607	0.8349
Campus	0	0.9977
Ethnicity	3.03	0.2193
**I was prescribed anxiolytic medication during the COVID-19 pandemic**		
Gender	1.4564	0.2275
Class year	1.6847	0.1943
Age (paired *t*-test)	−0.15	0.8803
Relationship status	2.0335	0.3618
Living situation	1.9758	0.5774
Campus	0.0233	0.8786
Ethnicity	2.8388	0.2419
**I saw a therapist for anxiety during the COVID-19 pandemic**		
Gender	2.3299	0.1269
Class year	0.2817	0.5956
Age (paired *t*-test)	−0.73	0.4668
Relationship status	2.5531	0.279
Living situation	6.2892	0.0984
Campus	2.9435	0.0862
Ethnicity	3.2118	0.2007
**I have felt more depressed during the COVID-19 pandemic**		
Gender	2.0596	0.1512
Class year	4.4623	0.347
Age (ANOVA)	0.24	0.8094
Relationship status	0.6533	0.7213
Living situation	4.1716	0.2435
Campus	2.2135	0.1368
Ethnicity	2.4549	0.2938
**I was prescribed antidepressant medication during the COVID-19 pandemic**		
Gender	1.5334	0.2156
Class year	4.0869	0.3944
Age (ANOVA)	−0.38	0.7037
Relationship status	1.1914	0.5512
Living situation	8.3449	0.0394
Campus	0.4169	0.5185
Ethnicity	2.7705	0.2503
**I saw a therapist for depression during the COVID-19 pandemic**		
Gender	0.9529	0.329
Class year	0.9785	0.3226
Age (paired *t*-test)	0.32	0.7514
Relationship status	2.3934	0.3022
Living situation	10.198	0.0169
Campus	1.1925	0.2748
Ethnicity	0.2782	0.8701
**I have felt hopeless, exhausted, or emotionally unresponsive during the COVID-19 pandemic**		
Gender	3.1214	0.0773
Class year	2.5484	0.636
Age (paired *t*-test)	0.83	0.4085
Relationship status	1.2527	0.5345
Living situation	3.5631	0.3127
Campus	0.6618	0.4159
Ethnicity	1.9685	0.3737

*indicates a significant *p*-value.

## Results

Out of the 596 students who were contacted, a total of 152 valid responses were received (25.5% response rate). Of these, 64.1% identified as female, 34.6% identified as male, and 1.3% reported a different gender identity. Participants were primarily White (75.8%), followed by Asian (14.4%), and mostly between the ages of 21–25 (50.3%) and 26–30 (43.1%). 77.8% of respondents were OMS-I students and 22.2% were OMS-II students. The lower response rate than expected was likely due to OMS-II students preparing for their medical board examinations, otherwise referred to as the USMLE STEP 1 Examination.

Most survey responses reflect RVU’s Colorado campus (68.0%) as compared to RVU’s Utah campus (32.0%). As this study was conducted by students on the RVU Colorado campus, the authors had limited access to communication with students on the RVU Utah campus *via* social media channels. This likely contributed to the disparity in response rate by campus. All participant demographics are listed in Table 3.

**Table 3:** Participant demographics.

Characteristic	No
Participants	152
**Gender**	
Female	97
Male	53
Non-binary	0
Prefer not to answer	2
**Class year**	
OMS I	118
OMS II	34
**Age range**	
21–25	77
25–30	66
30–35	7
35–40	2
**Relationship status**	
Single	50
In a relationship	69
Married	29
Engaged	4
**Living situation**	
Home/apartment with friends	51
With family	18
Alone	33
Significant other	46
Significant other w/ children	4
**Campus**	
CO	104
SU	48

**Figure 1:**
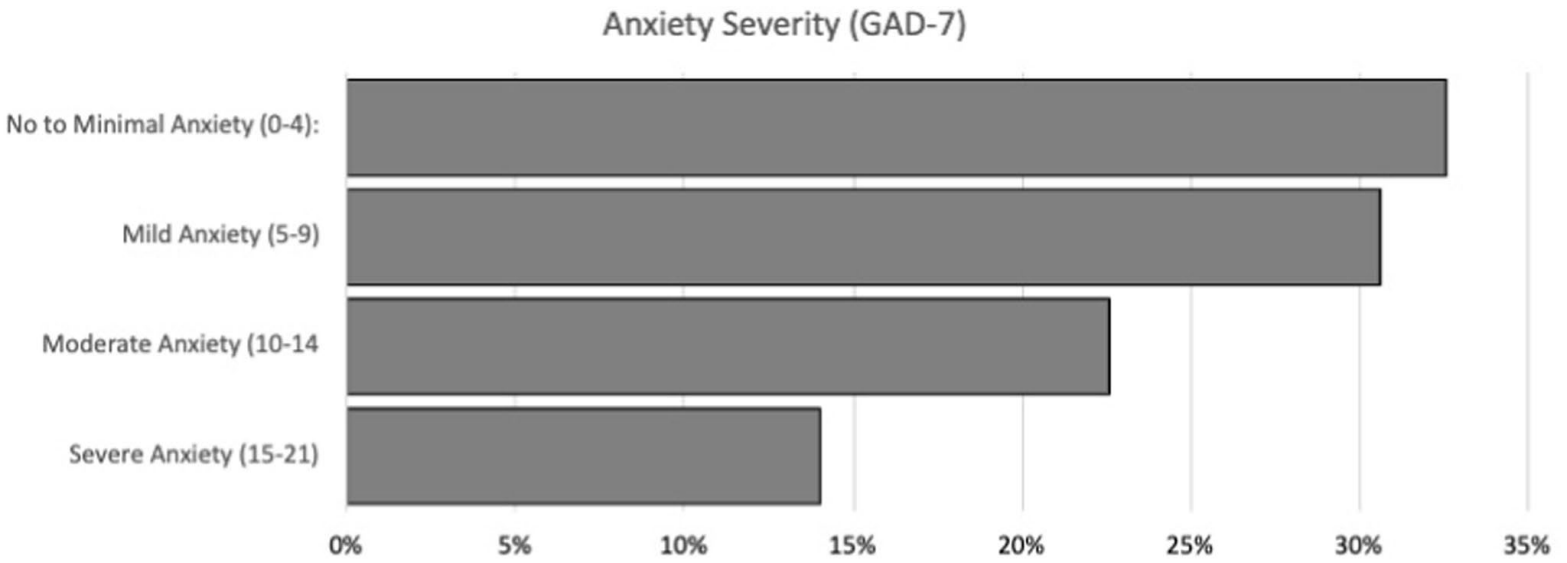
GAD-7 anxiety severity scores.

**Figure 2:**
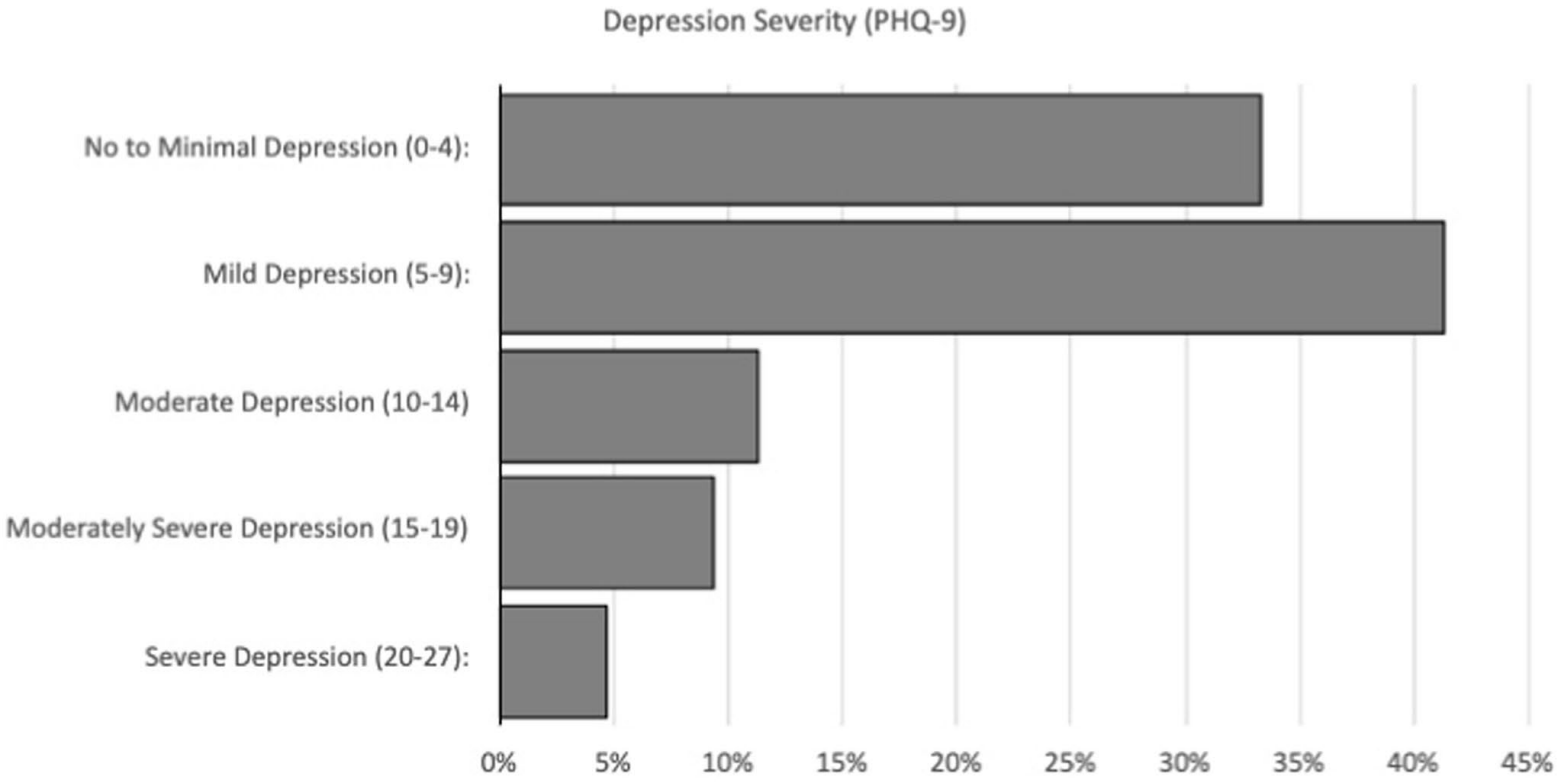
PHQ-9 depression severity scores.

As seen in Figure 1, GAD-7 scores revealed that 32.7% of participants screened negative for anxiety, while 30.7% screened positive for mild anxiety, 22.7% for moderate anxiety, and 14.0% for severe anxiety. Meanwhile, PHQ-9 results shown in Figure 2 indicated that 33.3% of respondents were experiencing no to minimal depression, while 41.3%, 11.3%, 9.3%, and 4.7% showed signs of mild, moderate, moderately severe, and severe depression, respectively.

**Figure 3:**
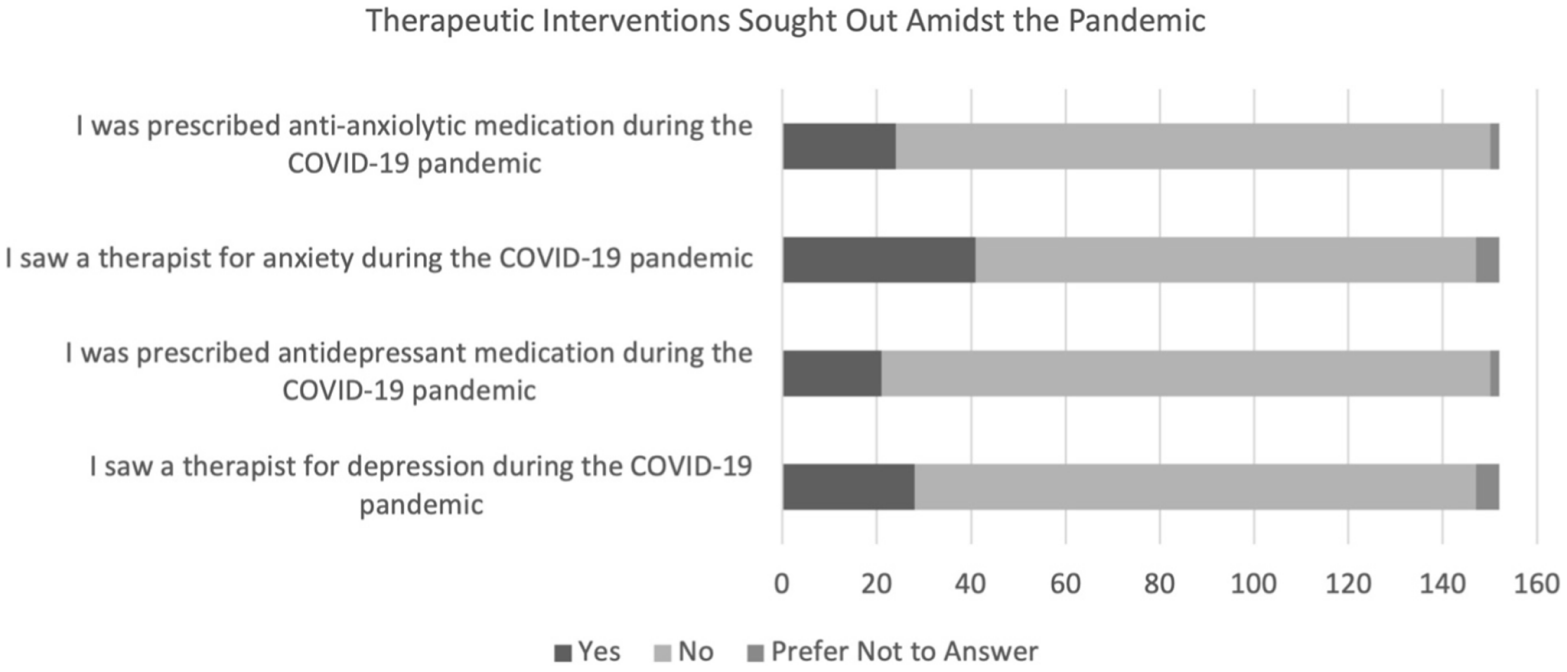
Therapeutic interventions sought out.

**Figure 4:**
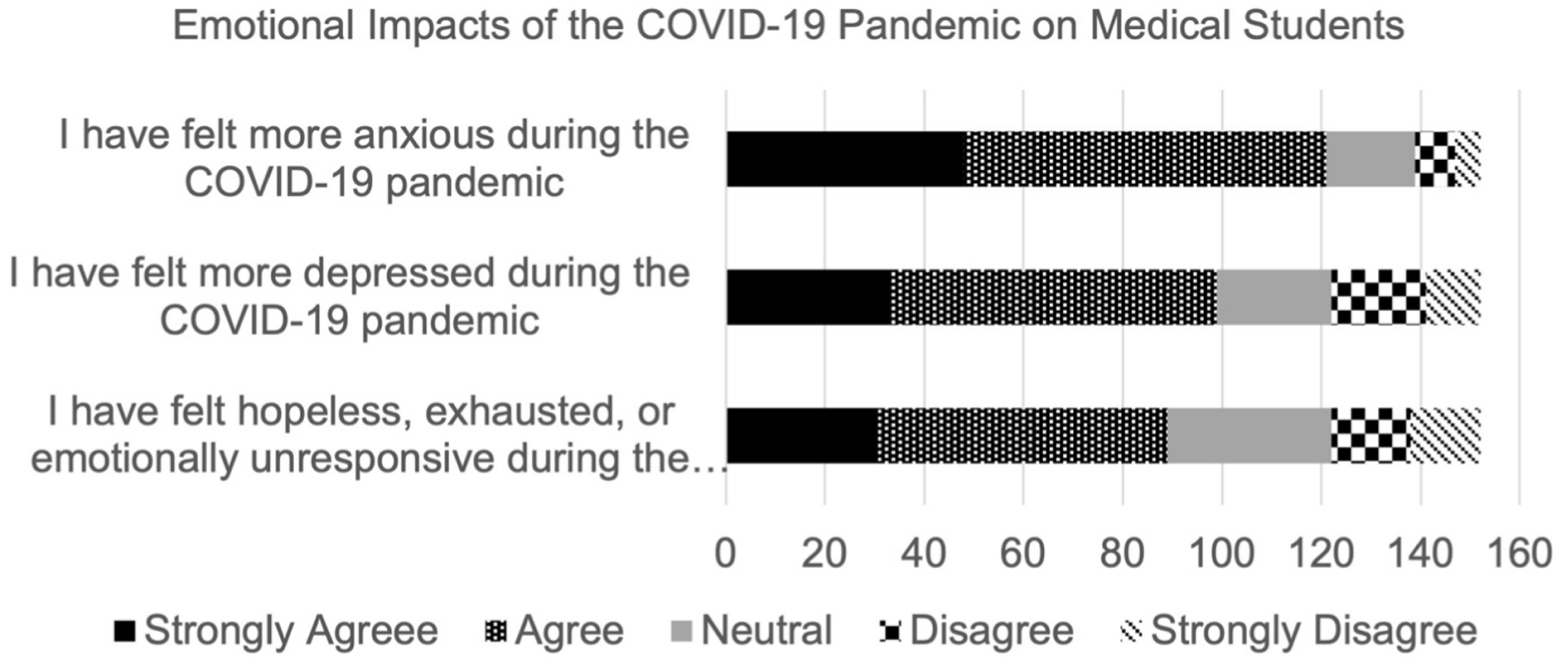
Emotional impact of the COVID-19 pandemic.

Most respondents agreed that they felt more anxious (79.6%), more depressed (65.1%), and hopeless, exhausted, or emotionally unresponsive (58.6%) during the pandemic. 15.8% and 13.8% of participants reported being prescribed anxiolytic and antidepressant medication during the pandemic, respectively; 27.0% reported seeing a therapist for anxiety and 18.4% reported seeing a therapist for depression. The majority of participants found it difficult to connect with others virtually (84.9%) and reported that social isolation due to the pandemic affected them negatively psychologically (67.8%). This is depicted in Figures 3, 4.

In our secondary analysis, we examined statistical differences between sub-groups by comparing responses to key questions, including GAD-7 and PHQ-9 scores which are shown in Tables 1, 2. While Table 1 reveals the association of Anxiety and Depression Scores and Demographic Factors, Table 2 illustrates the association of demographics with the Likert questions. Table 2 was constructed based on Likert question associations with demographics. Two groups were assembled based on Likert questionnaires. We grouped Likert responses 1 and 2, and 3–5 into their own separate categories to strengthen the data.

As seen in Table 1, women were significantly more likely than men to score higher on GAD-7 and report feeling more anxious during the COVID-19 pandemic (*p* = 0.0194). Students in a relationship were also significantly more likely to score higher on GAD-7, as compared to single and married students (*p* = 0.0244), shown in Table 1. Students living with friends were more likely to report feeling more anxious during the COVID-19 pandemic than those living with their parents, by themselves, or with their significant other (*p* = 0.3157). Students living with their significant others were least likely to report feeling anxious during the pandemic.

Additionally, OMS-II students scored significantly higher on PHQ-9, on average, than OMS-I students (*p* = 0.0142). Students of non-White and non-Asian ethnicities were more likely to report feeling depressed during the pandemic, although this finding likely needs to be investigated further due to the small sample sizes of Native American, Latino, Black, and mixed students.

Table 2 shows that students living with parents or with their significant others were significantly less likely to see a therapist for depression, as compared with students living by themselves or with their friends. Those living with their friends were most likely to see a therapist for depression (*p* = 0.0169). Lastly, students living with friends were more likely to be prescribed an antidepressant as compared to those living alone, with parents, or with a significant other (*p* = 0.0394). This data in Table 2 was analyzed and compared using the post-hoc Benjamini–Hochberg procedure.

## Discussion

The COVID-19 pandemic introduced a new norm for many around the globe and forced medical students to face unprecedented levels of uncertainty. Struggling to maintain balance with one’s personal life and rigorous academic requirements, COVID-19 has caused students to struggle with even more feelings of isolation, anxiety, stress, fear, and depression. The noteworthy changes in students’ mental health and wellbeing represent a pressing matter that the world needs to recognize. This study seeks to offer insight into the demographic factors that influence one’s mental health, the prevalence rates of anxiety and depression, the coping mechanisms utilized by current students at Rocky Vista University, and the impact of virtual education on mental health status.

### Anxiety

As mentioned previously, the global prevalence of anxiety among medical students, before the pandemic, was 33.8% (Tian-Ci Quek et al., 2019). When assessing the United States, a study conducted in 2016 screened 336 medical students for Generalized Anxiety Disorder, of which 20.3% of students screened positive using GAD-7 (Mousa et al., 2016). While our questionnaire did not assess a baseline level of anxiety among medical students, this pre-existing literature will be used to compare pre-pandemic and pandemic prevalence levels. A study conducted on 1,428 students from 40 United States medical schools during the pandemic reported a high prevalence (30.6%) of moderate to severe anxiety using the GAD-7 questionnaire, which is lower than the reported pre-pandemic global levels (Halperin et al., 2021). The results from that study are lower than those discovered in the RVUCOM questionnaire, with more than 36% of students experiencing moderate to severe anxiety. Even though the present study did not gather baseline data for the participants included, the results indicate that students were likely experiencing high levels of anxiety due to the rigors of medical school in general. Regarding patient demographics, higher GAD-7 scores were seen among female students (9.03 ± 5.23) and those in a relationship (9.50 ± 5.28). While previous studies have shown that anxiety is more prevalent among female medical students than males (Saltzman et al., 2021) we were surprised to find that those in a relationship were more anxious than those that were single or married; previous studies conducted on the general population during the pandemic have indicated that GAD-7 scores are higher among single individuals (Tian-Ci Quek et al., 2019). Our results might highlight anxiety that exists when navigating COVID-19 exposures for yourself and your significant other, as well as the stress created by quarantining with someone for prolonged periods of time. Previous studies have shown that couples who minimized isolation together were more likely to show resilience (Tian-Ci Quek et al., 2019). Being quarantined with a partner was almost inevitable during the pandemic, and was likely made worse by remaining in the house to study for an entire year. In order to support the data gathered by GAD-7, we included questions in the survey regarding the prescription of anxiolytic medications and therapist visitation. These questions revealed that 15.8% of students started an anxiolytic medication and 27.0% reported seeing a therapist for anxiety during the pandemic. To our knowledge, this is the first reported measure of these factors among medical students. A study was conducted among the general population with 2,739 United States participants aged 18 and older, which determined that 35.7% of survey takers reported increased use of anti-anxiety and sleep aid medications (Grigsby et al., 2022). The prevalence of anti-anxiety medication use among medical students, therefore, seems insufficient. These findings could indicate that medical students are finding inadequate mental health resources, are more hesitant to begin anxiety medications, or that they are less likely to seek help in general. More research needs to be done regarding the appropriate use of mental health aid among mental students to be able to conclude anything.

Our hypothesis, that the COVID-19 pandemic has had a negative effect on the mental health of pre-clinical medical students at Rocky Vista University which has led to worsened anxiety levels, was supported. The baseline prevalence of anxiety found among the global medical student population was 33.8% (Tian-Ci Quek et al., 2019) using the GAD-7, though the study did not report severity levels. The medical students at RVU had a combined prevalence of anxiety totaling 67.4%, ranging from mild (30.7%), moderate (22.7%), and severe (14%).

### Depression

The survey administered to RVUCOM students included the PHQ-9 instrument, which revealed that 25.34% of students scored above a 10 on the PHQ-9 scale, indicating a moderate to severe level of depression. This aligns with a cross-sectional study conducted on 40 US medical schools which determined that 24.3% of respondents scored positive for depression on the PHQ-9 scale (Halperin et al., 2021). Both studies have therefore shown that students are experiencing higher levels of depression during the COVID-19 pandemic compared to baseline.

After analyzing the data, significant correlations were observed pertaining to PHQ-9 scores. Higher PHQ-9 scores were seen among OMS II students (9.5 ± 6.39), which matches data collected through similar studies in the United States, indicating higher levels of depression among pre-clinical medical students (Christophers et al., 2021). This finding might reflect that OMS-II students are experiencing higher levels of depression due to the addition of the first US national medical school board examination, USMLE STEP 1, test preparation to their daily routines. Aside from the statistical difference seen between graduation classes, gender, ethnicity, age, relationship status, and living situation were not associated with the PHQ-9 score. Students’ living status had a significant correlation with therapy visits; 32.61% of students living with friends saw a therapist, compared with 21.88% of students living alone, 5.26% of students living with their family, and 10.42% of students living with a significant other. This correlation may be the result of students feeling encouraged by their friends to seek help and attend therapy sessions.

Prior research corroborates the results from this survey. In a large-scale study completed on 1,139 medical students from Washington and New York, researchers found that over two-thirds of medical students believed their mental health deteriorated during the pandemic, citing symptoms of anxiety and depression (Christophers et al., 2021). As discussed previously, medical students typically had higher baseline scores of anxiety and depression than the general public, and statistics have worsened over the last 2 years. As the world has generally acknowledged that healthcare workers are particularly vulnerable to developing mental health issues, especially during a pandemic, this research demonstrates that medical students share the same vulnerability (Schwenk et al., 2010). The current findings should guide medical schools, policy makers, and mental health professionals to implement systematic mental health screenings for medical students. Simple and effective screening methods could include GAD-7 and PHQ-9, as discussed in this article. New pandemics are likely to occur in the future, indicating that students will suffer the same fate if preventative measures are not taken. Such measures could include increasing access to mental health care, improving resources for coping, integrating in-person education back into this virtual world, acknowledging and having open discussions about mental health, and reducing stigma in medicine associated with mental health disorders.

This study revealed that 13.8% of students started an antidepressant and 18.4% saw a therapist for depression during the pandemic. To our knowledge, the existing literature regarding antidepressant use and the use of therapy for depression during COVID-19 is scarce. More research should be conducted to determine the trends in both factors. Our hypothesis, that the COVID-19 pandemic has had a negative effect on the mental health of pre-clinical medical students at Rocky Vista University which has led to worsened depression levels, was supported. Pre-pandemic levels of depression among medical students living in Michigan revealed a prevalence of 14.3%, ranging from moderate to severe depression, using the PHQ-9. The study at RVU revealed the prevalence of moderate to severe depression to be 25.3%; specific scores included moderate (11.3%), moderately severe (9.3%), and severe (4.7%) depression.

### Strengths

This study has several strengths. First, this study was conducted during the peak of the COVID-19 pandemic during a time when school was conducted virtually and social isolation was still prevalent. Second, to the researchers’ knowledge, this was the first study conducted on medical student mental health in Colorado. Additionally, this article was reinforced by its survey of OMSII students, since they had exposure to “normal” medical school conditions at RVU during their first year, prior to the pandemic. This allowed them to have a baseline standard of medical education and their mental health.

### Limitations

This study has several limitations. First, the survey respondents were primarily white, which is an accurate reflection of the student population at RVU. Studies have shown that communities of color in the United States were disproportionately affected by the pandemic; a study conducted revealed that people of color were 10 times more likely to meet the threshold criteria for depression, than their white counterparts (Pietromonaco and Overall, 2022). In order to generate data that more accurately reflects the United States population, future studies should aim to collect data from more diverse populations. Second, baseline PHQ-9 and GAD-7 scores were not collected and researchers had to gather data from the general United States medical student population to compare pre-pandemic and pandemic data, which might not accurately depict the prevalence at RVU. Third, collecting information through a self-reported questionnaire has inherent limitations due to response bias, and in order to make a clinical diagnosis of anxiety or depression, clinical assessment is necessary. Finally, there might be a systematic difference between individuals who volunteer to participate in a survey versus those who do not; because this survey was not a mandatory study, it might not accurately reflect RVU as a whole.

### Conclusion

While the pandemic has certainly worsened medical student mental health status, there are more studies being conducted that focus on this vulnerable population. Hopefully this data will be used to verify that preventative measures need to be taken in order to cushion the blow of a future potential pandemic. After conducting extensive research on this topic, within the United States and the global population, the researchers realized that this is a worldwide issue among medical students and the healthcare community alike. The literature regarding how medical students best utilize mental health services is limited, but previous studies have shown that even with a perceived need for help, students are not likely to seek treatment. A study among 2,868 medical students in Ohio in 2020 indicated that while 56% of students reported a perceived need for help, 34.6% of those students did not receive mental health treatment; barriers to service utilization included the lack of time, difficulty accessing services, and the stigma associated with having a mental health problem (Phillips et al., 2022). A similar study conducted at the University of Vermont indicated that the most common barriers to use of services were lack of time, lack of convenience, and concerns about what supervisors and other students would think (Rodriguez et al., 2017). Efforts should be made toward knocking down these barriers in a number of ways. For instance, schools could block off time in students’ schedules to allow them time to attend a counseling appointment, increase availability to include in person and online sessions with a variety of treatment options and services, and work to decrease personal and social stigma associated with mental health problems. The latter issue is likely related to the perceived negative impact on their careers, however, it is essential to outline how improving mental health can positively impact one’s ability to cope with academic rigors and to perform well in clinical settings. In a field that already reports high rates of mental health issues, suicide, and burn-out, it is up to the policy makers, medical schools, mentors, politicians, and general population, to take this matter seriously and impact actual change.

Healthcare workers live to care for others, so the question stands: who will take care of us?

## Data availability statement

The original contributions presented in the study are included in the article/Supplementary material, further inquiries can be directed to the corresponding author.

## Ethics statement

The studies involving human participants were reviewed and approved by Ethics Committee of Rocky Vista University. The patients/participants provided their written informed consent to participate in this study.

## Author contributions

DCP and VK contributed to conceptualization, data curation, formal analysis, funding acquisition, investigation of the project, methodology, project administration, resources, supervision, and original draft preparation, review, and editing. MB, MZ, and VB contributed to conceptualization, data curation, formal analysis, funding acquisition, investigation of the project, methodology, resources, and original draft preparation, review, and editing. MP contributed to formal analysis and software programming. RR contributed to formal analysis and review and editing. All authors contributed to the article and approved the submitted version.

## Funding

Granting was allocated by the Director of Research at Rocky Vista University for the amount of $3150.00. This was used as an incentive to reward participants.

## Conflict of interest

The authors declare that the research was conducted in the absence of any commercial or financial relationships that could be construed as a potential conflict of interest.

## Publisher’s note

All claims expressed in this article are solely those of the authors and do not necessarily represent those of their affiliated organizations, or those of the publisher, the editors and the reviewers. Any product that may be evaluated in this article, or claim that may be made by its manufacturer, is not guaranteed or endorsed by the publisher.
