# Supplementary material for: Impact of COVID-19 on Rocky Vista University medical students’ mental health: A cross-sectional survey
Source: Front Psychol. 2023 Feb 6;14:1076841. doi: 10.3389/fpsyg.2023.1076841 (PMC9939690; doi:10.3389/fpsyg.2023.1076841)
Supplement: Supplementary file 1 [file Data_Sheet_1.pdf]

STUDY RECRUITMENT BLURB

Dear Rocky Vista University Medical Student,

You are invited to participate in a research study about mental health. These questions are aimed at gathering data on how the COVID-19 pandemic has affected your experience of virtual medical education, prior expectations of medical education, perception of preparedness towards clinical skills, willingness to enter the medical field and pandemic related specialties, the impact that social isolation/virtual education has had on your mental health and any coping strategies you have found effective.

After completing the study, **you will receive a $10 Amazon gift card** as compensation for your time. We estimate participating in this study will take 10-15 minutes. You will also be informed of your anxiety and depression screening questionnaire results at the end of the survey. All responses will be kept **completely anonymous and confidential**, so please answer honestly. You will have the option of providing us with your RVU email address for receiving the gift card.

Use the link below to enroll in the study: (Link will appear once survey link has been constructed)

This study is being conducted by Dean Paz (dean.paz@rvu.edu), Manav Bains (manav.bains@rvu.edu), Morgan Zueger (Morgan.Zueger@RVU.edu), Victor Kuo (victor.kuo@rvu.edu), Varasiddimounish Bandi (Varasiddimounis.Bandi@rvu.edu) and our faculty mentor, Dr. Rebecca Ryznar, at (rryznar@rvu.edu).

If you have any questions about your rights as a research participant, please contact the Rocky Vista University IRB Compliance Administrator at 720-874-2481 or ldement@rvu.edu

Header that will be included on each page of the questionnaire: Need help now? Text "START" to 741-741 or call 1-800-273-TALK (8255)

Prior to beginning, please note the Mental Health Resources listed below. These will be listed again at the end of the survey. Please don’t hesitate to reach out if necessary!

- Utah Students: Dr. Kathy Killian-Harmon, LMFT; kkharmon@rvu.edu; Schedule an appointment https://www.rvu.edu/ut/student-affairs/mental-health-and-wellness/; urgent appointments available by request
- Colorado Students: Kären Robinson, LPC; krobinson@rvu.edu; Schedule an appointment https://www.rvu.edu/co/student-affairs/mental-health-and-wellness/; urgent appointments available by request and drop-in hours available Fridays 2-4 p.m. www.doxy.me/rvucounselingco
- StudentLinc 1-888-893-5462, contracted service for RVU Students, available 24/7/365
- National Suicide Prevention Lifeline 1-800-273-8255, available 24/7/365, routes to nearest crisis hotline
- SAMHSA Helpline 1-800-662-4357 (English & Spanish), available 24/7/365
- Crisis Textline, text HELLO to 741-741, available 24/7/365
- Trevor Project for LGBTQ+ Persons 1-866-488-7386 or Text START to 678-678
- Trans Lifeline 877-565-8860
- Veterans Crisis Line 1-800-273-8255, press 1 or Text 838255

Demographics

- What graduating class are you currently a part of?
    - OMS I
    - OMS II
    - OMS III
- What is your gender?
    - Male
    - Female
    - Non-binary
    - Prefer not to answer
- What is your ethnicity?
    - Multiple-option
- How old are you?
    - Free-response
- What is your religious background?
    - Multiple-option
- What is your relationship status?
    - Single
    - In a relationship
    - Married
    - Prefer not to say
- What is your living situation?
    - In a house or apartment with peers or friends
    - With my parents or other family member
    - By myself
- Which campus are you on?
    - SU/CO

Educational Changes

OMS III Specific

- *Boards*
    - If your USMLE/COMLEX exams were postponed due to COVID-19, approximately how many months were they postponed?
        - Free response
    - I changed my USMLE/COMLEX study habits due to COVID-19 related educational curriculum changes
    - I felt more prepared for USMLE/COMLEX exams due to COVID-19-related educational curriculum changes

All Classes

- *Impact on specialty choice*
    - What medical specialty were you most interested in pre-COVID? (open ended)
    - Because of COVID-19, my interest in ______ has grown (select all that apply):
        - Infectious Diseases
        - Pulmonology
        - Critical Care
        - Internal Medicine
        - Public Health
        - Epidemiology
        - Health Policy
        - Emergency Medicine
        - Other (open comments)
- *Impact of virtual education*
    - *Satisfaction*

        - I feel that faculty adequately used available resources to deliver course content
    - *Effectiveness of virtual education*
        - I am confident in my ability to study and learn effectively virtually during the pandemic
    - *Preference of education delivery method*
        - I prefer virtual classes to in-person classes
        - I prefer virtual labs to in-person labs
        - I prefer remote exams to in-person exams
        - I've had to identify new study strategies to succeed in class
    - *Preparedness for clinical rotations*
        - I felt equally prepared for clinical rotations due to COVID-19-related educational curriculum changes
        - Despite the inability to shadow clinicians, participate in volunteer opportunities and attend conferences in person, I feel virtual replacements have sufficiently prepared me to enter the medical field
- *Medical Education Expectations [7]* - Scale: Has been implemented during the pandemic, Should be implemented during the pandemic, Should be implemented after the pandemic
    - Podcasts
    - Online lectures
    - Independent collaborative work
    - Live broadcasts
    - Online chats with teachers
    - Online exams
    - Online collaborative work
    - Online study groups
    - Other (ie recommended literature readings) - open comment
- *Telehealth*
    - I have had a telehealth visit with a physician as a patient
    - I believe telehealth is and will continue to be an important part of healthcare
    - I found telehealth simulation through Standardized Patients to be useful and effective practice
    - I feel sufficiently prepared to conduct patient interviewing in-person
- *Mental Health*
    - *Motivation*
        - I am still excited about being a doctor
        - I have performed up to my expected academic standards
        - I've noticed an improvement in my studying performance during the pandemic
    - *Stress*
        - I've had to find new ways to cope with my stress due to educational demands during the pandemic
        - What has been your greatest stressor during the pandemic?
            - Financial stress
            - Health-related stress
            - Mental-health related stress
            - Social stress
            - Academic-related stress
            - Other (open comment)
    - *Anxiety*

        - I have had trouble concentrating on schoolwork during the pandemic
        - What about virtual medical education has caused you the greatest anxiety during the pandemic?
            - Technical difficulties
            - Does not fit my learning style
            - Less social contact with teachers and peers
            - Other (open comment)

Mental Health

Anxiety

- GAD-7
- I have felt more anxious during the pandemic.
- I was prescribed anti-anxiolytic medication during the COVID-19 pandemic.
- I saw a therapist for anxiety during the COVID-19 pandemic.

Depression

- PHQ-9
- I have felt more depressed during the pandemic
- I have felt hopeless, exhausted, or emotionally unresponsive during the pandemic
- I was prescribed antidepressant medication during the COVID-19 pandemic.
- I saw a therapist for depression during the COVID-19 pandemic.

Factors associated with COVID-19

- *Concern about the pandemic*
    - I have had frequent thoughts of being infected by COVID-19 during the pandemic
    - I am concerned a family member or loved one will get COVID-19
    - I am concerned that I may spread COVID-19 to others
- *Willingness to assist with pandemic*
    - I am confident in my ability to assist in a crisis
    - I would be willing to offer support during the COVID-19 pandemic
    - I would be willing to treat patients without COVID-19
    - I would be willing to treat patients with COVID-19
- *Level of knowledge about the pandemic*
    - I consider myself knowledgeable about the pandemic
    - I stay up-to-date on the most recent news about the pandemic
    - I adhere to CDC and RVU pandemic control guidelines
    - On average, how much time do you spend daily following COVID-19?
        - None or almost none
        - Less than 15 minutes
        - Between 15-30 minutes
        - Between 30-45 minutes
        - Between 45 minutes to 1 hour
        - Greater than 1 hour
- *Vaccine*
    - Life will return to pre-pandemic “normal” after enough people have received the vaccine
    - I am nervous about the long-term side effects of the COVID-19 vaccine due to inadequate long-term research
    - I feel hopeful about the future because of the availability of the vaccine

Behavioral Changes

- *Sleep*
    - I have felt more rested during the pandemic
    - How many hours a night do you sleep on average?

    - How many hours a night did you sleep on average before the pandemic?
- *Screen time*
    - I spent more time looking at screens during the pandemic
- *Exercise*
    - How many hours a week do you exercise on average?
    - How many hours a week did you exercise on average before the pandemic?
- *Diet*
    - I have eaten healthier during the the pandemic
- *Alcohol consumption*
    - How many drinks do you consume weekly on average? Assume 1 beer/glass of wine = 1 drink and 1 shot = 1 drink
    - How many drinks do you consume weekly on average before the pandemic?
- *Social Contact*
    - Social isolation due to the pandemic has really negatively affected me psychologically
    - I have found it difficult to connect with others virtually
    - I have found it difficult to study on my own
    - I have found it difficult to find people to study with

Coping Strategies

- *General*
    - Rocky Vista University has provided sufficient behavioral coping resources during the pandemic
    - I would like more information about strategies to cope with stress and anxiety
    - I know where and how to access mental health support when I need it
    - What have you found to be a useful coping strategy during the pandemic? (check all that apply)
        - Yoga
        - Meditation
        - Religious activities
        - Physical exercise
        - Virtual social interaction
        - Therapy
        - Pets
        - Spending time with family (in-person or virtually)
        - Greenery/Gardening
        - Food
        - Government resources (ie CDC guidance, stimulus check)
        - Other (fill in the blank)

Thank you for completing the survey.

Your screening scores for anxiety and depression are:

- [Insert GAD-7 score and screened anxiety level]
- [Insert PHQ-9 score and screened depression level]

For more information regarding your Generalized Anxiety Disorder (GAD-7) and Patient Health Questionnaire (PHQ-9) results, please visit the following link.

Please note the Mental Health Resources listed below, and don't hesitate to reach out if necessary.

- Utah Students: Dr. Kathy Killian-Harmon, LMFT; kkharmon@rvu.edu; Schedule an appointment https://www.rvu.edu/ut/student-affairs/mental-health-and-wellness/; urgent appointments available by request
- Colorado Students: Kären Robinson, LPC; krobinson@rvu.edu; Schedule an appointment https://www.rvu.edu/co/student-affairs/mental-health-and-wellness/; urgent appointments available by request and drop-in hours available Fridays 2-4 p.m. www.doxy.me/rvucounselingco
- StudentLinc 1-888-893-5462, contracted service for RVU Students, available 24/7/365
- National Suicide Prevention Lifeline 1-800-273-8255, available 24/7/365, routes to nearest crisis hotline
- SAMHSA Helpline 1-800-662-4357 (English & Spanish), available 24/7/365
- Crisis Textline, text HELLO to 741-741, available 24/7/365
- Trevor Project for LGBTQ+ Persons 1-866-488-7386 or Text START to 678-678
- Trans Lifeline 877-565-8860
- Veterans Crisis Line 1-800-273-8255, press 1 or Text 838255

References

Xiong J, Lipsitz O, Nasri F, et al. Impact of COVID-19 pandemic on mental health in the general population: A systematic review. *J Affect Disord*. 2020;277:55-64. doi:10.1016/j.jad.2020.08.001

Liu J, Zhu Q, Fan W, Makamure J, Zheng C, Wang J. Online Mental Health Survey in a Medical College in China During the COVID-19 Outbreak. *Front Psychiatry*. 2020;11. doi:10.3389/fpsyt.2020.00459

Xiao H, Shu W, Li M, et al. Social Distancing among Medical Students during the 2019 Coronavirus Disease Pandemic in China: Disease Awareness, Anxiety Disorder, Depression, and Behavioral Activities. *Int J Environ Res Public Health*. 2020;17(14). doi:10.3390/ijerph17145047

Ye W, Ye X, Liu Y, et al. Effect of the Novel Coronavirus Pneumonia Pandemic on Medical Students' Psychological Stress and Its Influencing Factors. *Front Psychol*. 2020;11. doi:10.3389/fpsyg.2020.548506

Meo SA, Abukhalaf AA, Alomar AA, Sattar K, Klonoff DC. COVID-19 Pandemic: Impact of Quarantine on Medical Students' Mental Wellbeing and Learning Behaviors. *Pak J Med Sci*. 2020;36(COVID19-S4):S43-S48. doi:10.12669/pjms.36.COVID19-S4.2809

Xie L, Luo H, Li M, Ge W, Xing B, Miao Q. The immediate psychological effects of Coronavirus Disease 2019 on medical and non-medical students in China. *Int J Public Health*. Published online September 10, 2020:1-9. doi:10.1007/s00038-020-01475-3

Loda T, Löffler T, Erschens R, Zipfel S, Herrmann-Werner A. Medical education in times of COVID-19: German students' expectations - A cross-sectional study. *PLoS One*. 2020;15(11):e0241660. doi:10.1371/journal.pone.0241660

Khasawneh AI, Humeidan AA, Alsulaiman JW, et al. Medical Students and COVID-19: Knowledge, Attitudes, and Precautionary Measures. A Descriptive Study From Jordan. *Front Public Health*. 2020;8. doi:10.3389/fpubh.2020.00253

Wu S, Li Z, Li Z, et al. The mental state and risk factors of Chinese medical staff and medical students in early stages of the COVID-19 epidemic. *Compr Psychiatry*. 2020;102:152202. doi:10.1016/j.comppsych.2020.152202

Dubey S, Biswas P, Ghosh R, et al. Psychosocial impact of COVID-19. *Diabetes Metab Syndr*. 2020;14(5):779-788. doi:10.1016/j.dsx.2020.05.035

Shin TH, Klingler M, Han A, et al. Efficacy of Virtual Case-Based General Surgery Clerkship Curriculum During COVID-19 Distancing. *MedSciEduc*. Published online November 10, 2020. doi:10.1007/s40670-020-01126-5

Martin A, Blom IM, Whyatt G, Shaunak R, Viva MIF, Banerjee L. A Rapid Systematic Review Exploring the Involvement of Medical Students in Pandemics and Other Global Health Emergencies. *Disaster Medicine and Public Health Preparedness*. Published online undefined/ed:1-13. doi:10.1017/dmp.2020.315
